# Tautomeric equilibrium and spectroscopic properties of 8-azaguanine revealed by quantum chemistry methods

**DOI:** 10.1007/s00249-023-01672-x

**Published:** 2023-07-28

**Authors:** Maciej Maciejczyk, Maciej Pyrka

**Affiliations:** Department of Physics and Biophysics, University of Warmia and Mazury in Olsztyn, Oczapowskiego 4, 10-719 Olsztyn, Poland

**Keywords:** Tautomeric equilibrium, Nucleobases, Vertical transition energies, 8-Azaguanine

## Abstract

8-azaguanine is a triazolopyrimidine nucleobase analog possessing potent antibacterial and antitumor activities, and it has been implicated as a lead molecule in cancer and malaria therapy. Its intrinsic fluorescence properties can be utilized for monitoring its interactions with biological polymers like proteins or nucleic acids. In order to better understand these interactions, it is important to know the tautomeric equilibrium of this compound. In this work, the tautomeric equilibrium of all natural neutral and anionic compound forms (except highly improbable imino-enol tautomers) as well as their methyl derivatives and ribosides was revealed by quantum chemistry methods. It was shown that, as expected, tautomers protonated at positions 1 and 9 dominate neutral forms both in gas phase and in aqueous solution. 8-azaguanines methylated at any position of the triazole ring are protonated at position 1. The computed vertical absorption and emission energies are in very good agreement with the experimental data. They confirm the validity of the assumption that replacing the proton with the methyl group does not significantly change the positions of absorption and fluorescence peaks.

**Supplementary Information:**

The online version contains supplementary material available at 10.1007/s00249-023-01672-x.

## Introduction

Proto-tautomeric equilibrium is one of the fundamental phenomena that determines interactions between nucleic acid bases, the formation of three-dimensional structures of nucleic acids, and interactions of nucleic acid bases with proteins. Tautomeric protons are very often involved in intra- or inter-molecular hydrogen bonding, stabilizing the spatial structure of biomolecules. Tautomeric equilibrium of small molecules binding to the active sites of enzymes is particularly important because it affects the formation of protein–ligand hydrogen bonding patterns, and is therefore one of the key factors determining the most probable binding pose of the ligand. It is worth noting that destabilization of the lowest-energy tautomer that affects the shape of the Watson–Crick edge of any natural nucleobase would break classical nucleobase pairing, and life could not exist in its present form. The tautomeric equilibrium of nucleobases and their derivatives have been extensively studied by both computational (Sabio et al. 1990; Kwiatkowski and Leszczyński 1990; Alyoubi and Hilal 1995; Gorb and Leszczynski 1998b, a; Contreras and Madariaga 1998; Marchand-Geneste and Carpy 1999; Mennucci et al. 2001; Hanus et al. 2003; Blas et al. 2004; Piacenza and Grimme 2004; Kim et al. 2007; Fogarasi 2008; Kosenkov et al. 2009; Pancucci et al. 2011; Raczyńska et al. 2013; Pyrka and Maciejczyk 2015, 2016, 2020; Raczyńska 2017; Eberlein et al. 2020) and experimental (Chenon et al. 1975; Sepiol et al. 1976; Seela et al. 1995; Włodarczyk et al. 2004; Choi and Miller 2006; Wierzchowski et al. 2012, 2013; Karalkar et al. 2017) methods and reviewed by (Shukla and Leszczynski 2013).

On the other hand, it has been known since the early 70s that canonical nucleobases exhibit very low fluorescence emission quantum yields (Daniels and Hauswirth 1971; Morgan and Daniels 1979), and chemically modified nucleobase analogs are necessary in order to study biochemical processes involving nucleobases with fluorescence spectroscopy methods. An important class of fluorescent nucleobase analogs are purines, in which the C8 carbon of the triazole ring is replaced by nitrogen. The photochemistry of four fluorescent nucleobase aza analogs in acetonitrile solution was studied by (Kobayashi et al. 2009) by means of absorption and emission spectra and computational methods. These molecules are isosteric with natural nucleobases, but exhibit measurable emission in both neutral and ionic forms.

8-Azaguanine (8AG, PubChem CID—135403646, also known as pathocidin) is a triazolopyrimidine nucleobase analog possessing potent antibacterial and antitumor activities. It can replace guanine in RNA, resulting in cellular toxicity (Bergquist and Matthews 1962; Rivest et al. 1982a), and its incorporation in the m-RNA of a tumor cell inhibits protein synthesis (Zimmerman and Greenberg 1965). 8AG also interacts with 43S and 80S initiation complexes, causing inhibition of the protein translation process, and competes with guanine for incorporation into tRNA. Therefore, it has been implicated as a lead molecule in cancer (Nelson et al. 1975; Rivest et al. 1982b) and malaria therapy (Keough et al. 2006). It is a natural product, bearing a rare nitrogen-rich heterocycle (Kumar and Kaur 2014) with antineoplastic and antimetabolite activity, which was first found as a secondary metabolite in *Streptomyces albus* var. *pathocidicus* (Anzai et al. 1961). Recently, a biosynthetic gene cluster responsible for the synthesis of 8AG in *Streptomyces* was located, and a complete biosynthetic pathway of 8AG was proposed (Zhao et al. 2020; Hou et al. 2020). Intrinsic fluorescence properties of 8AG can be utilized for monitoring the reaction of ribosylation/phosphorolysis catalyzed by the purine nucleoside phosphorolysis (PNP) enzyme (Wierzchowski et al. 2004, 2005, 2013). A detailed mechanism of excited-state relaxation of 8AG in acetonitrile solution was also investigated by means of the MS-CASPT2 method (Sanches De Araújo and Borin 2019). The fluorescence of 8AG is also pH-dependent, and therefore it was used to probe the ionization states of nucleobases in structured RNAs (Da Costa et al. 2007). The replacement of the C8 carbon of Guanine should not directly influence both Watson–Crick and Hoogsteen interfaces, although it can possibly change the tautomeric equilibrium of the new compound compared to the unmodified Guanine. The most recent computational study of tautomeric equilibrium of 8AG performed by Contreras and Madariaga (Contreras and Madariaga 1998) showed that in water, the most probable are tautomers of 8AG protonated at positions 1 and 7, but the other two tautomeric forms (amino-enol form and amino-oxo protonated at positions 1 and 9) investigated by them are also significantly populated. The domination of A17 tautomer in water solution was confirmed by comparison of the ultraviolet resonance Raman spectra with results of DFT computations (Gogia and Puranik 2014), but it seems to contradict conclusions drawn from comparison of UV–Vis spectra of natural 8AG and its methylated forms (Wierzchowski et al. 2014), which clearly points to A19 tautomer.

The aim of this publication is to verify these relatively old results and extend the tautomeric picture of 8AG to the other amino-oxo, amino-enol and imino-oxo forms. In our previous publication (Pyrka and Maciejczyk 2016), it was shown that methylation of 8-aza-iso-guanine surprisingly significantly stabilizes enol tautomers in water solution. Therefore, in this work, also the influence of methylation of 8AG at various positions of the triazole ring on tautomeric equilibrium was investigated. Moreover, the vertical excitation (${E}^{vert-a}$) and the emission (${E}^{vert-f}$) energies of all tautomers (both methylated and nonmethylated) were calculated and compared to the available experimental data. This study can also serve as a mini-benchmark of three selected methods applied to various forms of the 8AG molecule and aims to answer the question of which tautomeric form is responsible for the fluorescence spectrum of 8AG.

## Methods

Prototropy of nucleobases and their derivatives were already extensively studied by ab initio methods (Shukla and Leszczynski 2013). In our previous publications (Pyrka and Maciejczyk 2015, 2016), both hybrid DFT-BHandHLYP functional (Becke 1993) combined with triple zeta basis set (Dunning 1989) and GAUSSIAN composite G3 (Curtiss et al. 1998) and G4 (Curtiss et al. 2007) methods were applied to tackle the problem of tautomeric equilibrium of 2,6-diamino-azapurine and 8-aza-isoguanine (also in the methylated form). Also, some preliminary computations for 8AG were performed using the BHandHLYP/cc-pvtz level of theory in order to set up the ligand–receptor system for molecular dynamics simulations (Pyrka and Maciejczyk 2020). It was shown that pure DFT methods generate reliable molecular geometries, but the corresponding energetics is wrong (Sonnenberg et al. 2009), and inclusion of 50% of Hartree–Fock exchange in the BHandHLYP functional resolves the problem, leading to energies comparable to those obtained with the QCISD(T) method (Piacenza and Grimme 2004).

All calculations were performed using the Gaussian16 package (Frisch et al. 2016). The primary method applied for the determination of populations of tautomers in the investigated system was a combination of BHandHLYP hybrid functional and augmented double zeta basis set (aug-cc-pvdz). The influence of basis set was investigated by the application of augmented triple zeta (aug-cc-pvtz) with the same functional. The results were compared to those obtained with the Gaussian G3 composite method. The geometries of all investigated tautomers were optimized in the ground state, both in the gas phase and in water solution, using at least tight convergence criteria. Then vibrational analysis was performed in order to check whether the investigated system had reached an energy minimum. Zero-point energies and thermal corrections (including contributions from translational, rotational motions, and internal vibrations) to free energies were computed in order to determine entropies ($\Delta S$), enthalpies ($\Delta H$), and Gibbs free energies of formation ($\Delta G$) at the temperature of 300 K (McQuarrie and Simon 1999; Ochterski 2000). The presence of solvent was emulated by the IEF-PCM model (Miertus et al. 1981; Miertus and Tomasi 1982). The geometries of all molecules were also optimized in the water solution. The free energy of solvation was estimated as $\Delta {\Delta G}_{solv}=\Delta {G}_{wat}-\Delta {G}_{vac}$, where $\Delta {G}_{wat}$ and $\Delta {G}_{vac}$ are the free energies of tautomer formation in water and vacuum, respectively. Populations of tautomers were estimated according to the Boltzmann distribution.

Vertical transition energies in the gas phase are defined as the difference between ground state (GS) and excited state (ES) energies calculated for the optimal GS geometry (${E}^{vert-a}$—vertical excitation or absorption energy) or optimal ES geometry (${E}^{vert-f}$—vertical emission or fluorescence energy). When the investigated molecule is surrounded by the solvent (e.g., water molecules), both GS and ES energies must be corrected by the value of the energy of solvation. As the electronic excitation and de-excitation are instantaneous, compared to the time of relaxation of solvent molecules, both equilibrium and non-equilibrium solvation models must be applied. The former model assumes that both the electronic cloud and the positions of the atomic nuclei of the solvent molecules had enough time to relax to reach equilibrium. This solvation model is used for the approximation of initial states—GS for the vertical excitation and ES for the vertical emission. The non-equilibrium solvation model assumes that only the electronic cloud of solvent molecules is able to instantaneously adjust to a very fast change of the electronic structure of the solute molecule. This solvation model is used in the approximation of final states—ES for vertical excitation and GS for vertical emission processes (Jacquemin and Adamo 2016). Equilibrium and non-equilibrium solvation energies can be significantly different, especially in polar solvents (Tomasi et al. 2005).

The vertical transition energies of all investigated tautomers were computed using the TDDFT method (Runge and Gross 1984; Gross and Kohn 1990; Van Leeuwen 2001; Casida and Huix-Rotllant 2012) implemented in GAUSSIAN software (Stratmann et al. 1998) with three hybrid functionals: B3LYP (Lee et al. 1988; Becke 1988), PBE0 (Perdew et al. 1996), and M06 (Zhao et al. 2008), which were shown to produce the best agreement with experimental data for valence excited states (Leang et al. 2012; Laurent and Jacquemin 2013). These functionals were combined with the aug-cc-pvdz basis set, which was shown to be sufficient for computations of vertical valence transitions (Jacquemin and Adamo 2016). It was also shown that for medium-sized molecules, the vertical excitations do not vary much with the size of the basis set if both polarization and diffuse functions are included (Jacquemin et al. 2012).

## Results and discussion

**Fig. 1:**
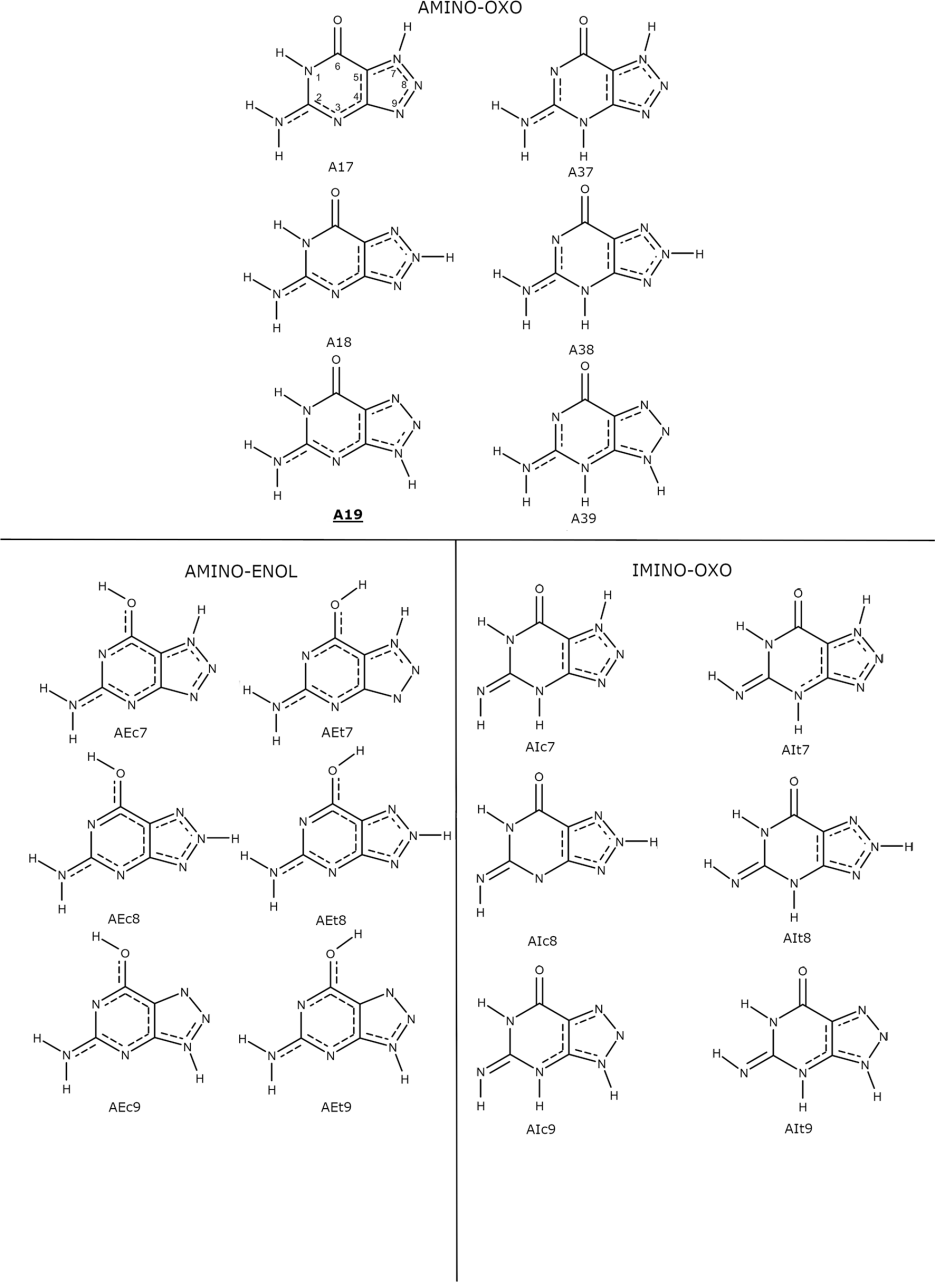
All neutral tautomeric forms of 8-azaguanine considered in this publication. The populations of imino-enol forms were assumed to be negligible. The assignment of atom numbers is presented in A17 tautomer. The dominant tautomer is marked with a boldface type caption. Bond-orders are marked according to Natural Bond-Orbital (NBO) analysis

**Fig. 2:**
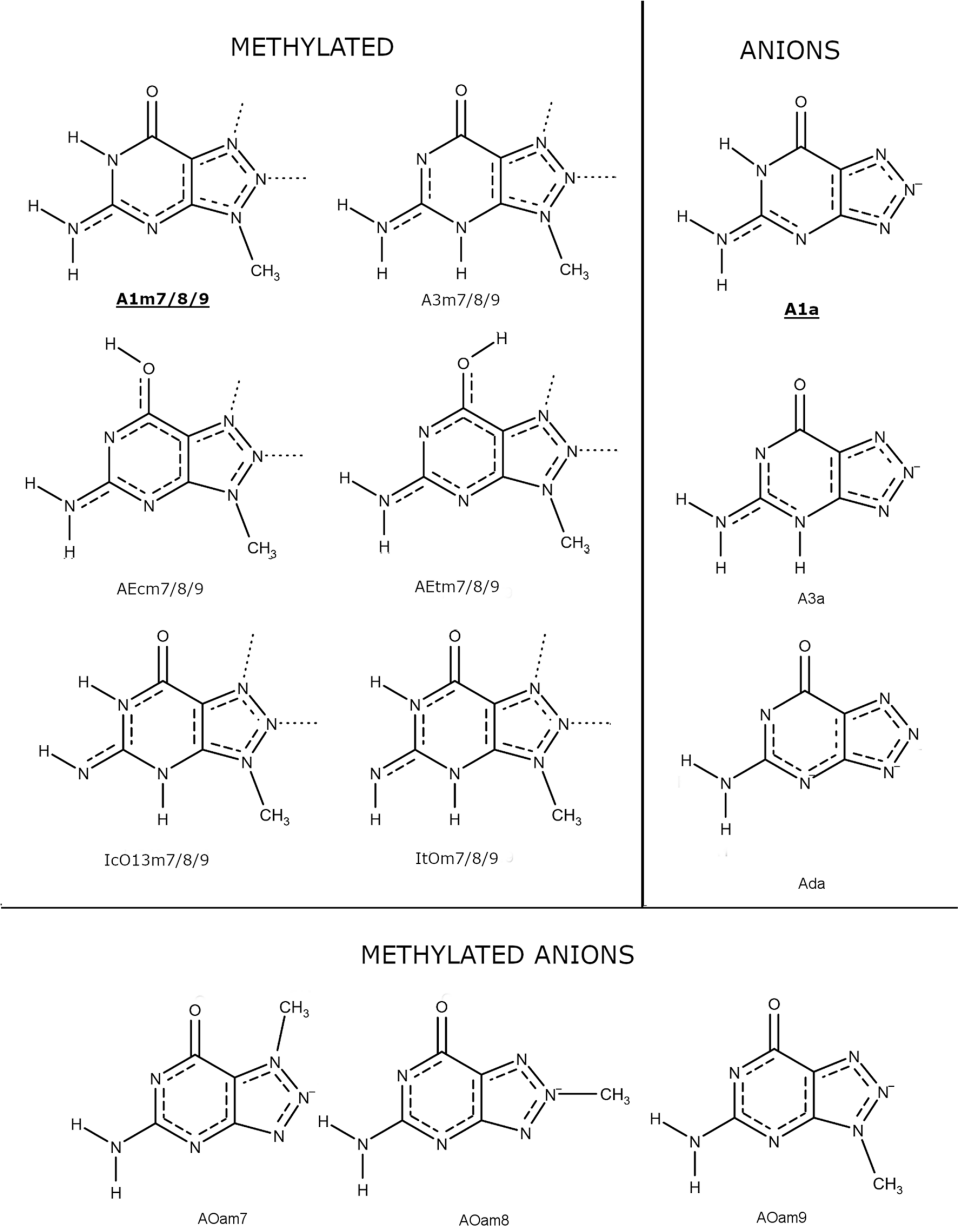
All methylated and/or ionic forms of 8-azaguanine considered in this publication. Alternative methyl positions are marked with a dotted line. The dominant tautomers of methylated and two mono-anionic forms are marked with bold-face type captions. Bond-orders are marked according to NBO analysis

All natural forms of the investigated tautomers are shown in Fig. 1. In physiological conditions, most nucleobases exist in oxo-amino forms, although in our recent publication (Pyrka and Maciejczyk 2016), it was shown that some enol-amino and oxo-imino forms of 8aza-isoguanine have relatively low free energies and should be considered minor species in water solution. Therefore, besides oxo-amino, also enol-amino and oxo-imino forms were also included in the analysis. Based on our study of 8-aza-iso-guanine (Pyrka and Maciejczyk 2016), enol-imino forms were assumed to have high energy and therefore excluded from our calculation. Also, molecules methylated on the triazole ring at positions 7, 8, and 9 were considered in both neutral and monoanionic forms (see Fig. 2). For these compounds, the tautomeric proton was located on its pyrimidine ring. Finally, two monoanions and one dianion shown in Fig. 2 were also investigated.

### Tautomeric equilibrium

The relative Gibbs free energies of all non-methylated tautomeric forms in the gas phase are shown in Table 1. A detailed decomposition of free energies and populations of tautomers is shown in Table S1 of the SI. All applied methods predicted the following order of the most populated tautomers A19 → A18 → AEc9 → AEt9 → A17 and pointed to A19 as the most stable tautomeric form in line with our previous studies of this compound performed with the BHandHLYP/cc-pvtz method (Pyrka and Maciejczyk 2020). There are four tautomeric forms which are energetically close to A19 in the gas phase—two amino-oxo forms (A18 and A17) and two amino-enol forms (A9c and A9t). Other tautomeric forms are practically non-existent in the gas phase, with free energy at least 5 kcal/mol higher than free energy of the dominant A19 form. Transferring the molecules from vacuum to water solution destabilizes the energetically closest tautomeric forms with respect to A19, as can be seen in Table 2. This effect is caused by the worse solvation of the above-mentioned tautomers in comparison to the A19 form, which in turn is related to their smaller dipole moments. It should be noted that only A39 tautomer has a significantly bigger dipole moment than A19, and its free energy of solvation is significantly lower (~ 9 kcal/mol). Nevertheless, A39 remains marginally populated in water because of its very high relative free energy in the gas phase.

**Table 1:** Relative Gibbs free energies in the gas phase and in water and relative energies of solvation determined for various 8AG tautomers with respect to the lowest-energy A19 tautomer. Three quantum chemistry methods were applied. Hybrid BHandHLYP/aug-cc-pvdz (BHH/D) and BHandHLYP/aug-cc-pvtz (BHH/T) and composite Gaussian G3. All energies are in kcal/mol. Dipole moments (p) of tautomers in solution obtained with the BHandHLYP/aug-cc-pvdz method are shown in the third column from the end. The last two columns show charges located on triazole and pyrimidine rings obtained with the same method

Tautomer		Gas phase			Water			Solvation			*P* (D)	Charges	
		BHH/D	BHH/T	G3	BHH/D	BHH/T	G3	BHH/D	BHH/T	G3		*Q*_T_	*Q*_P_
Amino-oxo	A17	2.5	2.7	5.1	4.4	4.9	6.3	1.8	2.2	1.2	7.1	− 0.06	0.02
	A18	0.8	1.0	2.6	3.0	3.5	4.4	2.2	2.6	1.7	5.2	− 0.04	− 0.07
	A19	0.0	0.0	0.0	0.0	0.0	0.0	0.0	0.0	0.0	11.4	− 0.08	− 0.05
	A37	8.4	8.3	11.5	7.5	7.5	10.6	− 0.8	− 0.7	− 0.9	7.8	− 0.05	− 0.10
	A38	9.2	9.1	11.2	7.0	7.2	8.9	− 2.2	− 1.9	− 2.4	11.7	− 0.03	− 0.10
	A39	17.9	17.8	19.5	9.2	9.3	10.5	− 8.7	− 8.5	− 9.0	17.0	− 0.06	− 0.09
Amino-enol	AEc7	6.7	7.2	11.7	12.4	13.3	16.5	5.7	6.1	4.8	7.5	− 0.06	− 0.08
	AEt7	13.4	13.9	19.9	15.7	16.6	20.6	2.3	2.7	0.8	6.4	− 0.06	0.00
	AEc8	5.3	5.8	10.8	12.1	13.0	16.4	6.8	7.2	5.6	2.8	− 0.05	0.01
	AEt8	6.9	7.4	13.8	13.6	14.6	18.8	6.8	7.1	5.0	2.7	− 0.04	0.00
	AEc9	0.8	1.2	3.2	7.6	8.5	9.6	6.9	7.3	6.5	6.3	− 0.08	0.03
	AEt9	1.5	1.5	4.7	9.0	10.2	11.7	7.5	8.8	7.0	3.5	− 0.07	0.02
Imino-oxo	AIc7	9.5	9.4	12.2	14.5	17.7	16.5	4.9	8.4	4.3	2.0	− 0.03	− 0.05
	AIt7	9.4	9.3	12.0	14.2	14.5	16.2	4.8	5.2	4.2	3.1	− 0.03	− 0.05
	AIc8	7.1	7.0	8.8	12.0	12.3	13.1	4.9	5.3	4.3	7.9	− 0.01	− 0.05
	AIt8	7.5	7.4	9.1	11.9	12.1	12.8	4.4	4.8	3.7	1.7	− 0.01	− 0.13
	AIc9	13.1	12.9	13.7	13.0	13.2	13.3	− 0.1	0.3	− 0.4	6.8	− 0.04	− 0.04
	AIt9	14.7	14.5	15.6	13.2	13.4	13.5	− 1.5	− 1.1	− 2.1	11.0	− 0.04	− 0.12

**Table 2:** Relative Gibbs free energies in the gas phase and water, and relative solvation free energies obtained for methylated forms of 8AG. For each methylated molecule, the most populated is the A1 form, protonated at position 1. For a description of methods, see the caption of Table 1. All energies are provided in kcal/mol

Tautomer		Gas phase			Water			Solvation			*P* (D)	Charges	
		BHH/D	BHH/T	G3	BHH/D	BHH/T	G3	BHH/D	BHH/T	G3		*Q*_T_	*Q*_P_
9 M	A1	0.0	0.0	0.0	0.0	0.0	0.0	0.0	0.0	0.0	11.7	− 0.08	0.08
	A3	18.0*	18.1	20.0	10.4*	9.4	10.9	− 7.6*	− 8.8	− 9.0	17.6*	− 0.03*	− 0.16*
	AEc	1.1	1.4	3.4	8.3	8.0	9.6	7.3	6.6	6.2	6.3	− 0.07	− 0.03
	AEt	1.5	1.9	5.1	9.6	9.4	11.6	8.1	7.5	6.5	4.0	− 0.06	0.01
	AIc	13.0	13.0	15.0	14.0	13.4	14.0	1.0	0.4	− 1.0	8.5	− 0.02	− 0.05
	AIt	14.6	14.6	16.9	14.0	13.5	14.2	− 0.6	− 1.1	− 2.8	11.7	− 0.01	− 0.05
8 M	A1	0.0	0.0	0.0	0.0	0.0	0.0	0.0	0.0	0.0	4.4	− 0.02	0.00
	A3	8.5	8.3	8.9	3.8	3.5	4.6	− 4.7	− 4.8	− 4.2	11.8	0.00	− 0.05
	AEc	4.3	4.7	7.9	8.8	9.2	11.8	4.5	4.5	3.9	2.0	− 0.02	− 0.01
	AEt	6.1	6.4	10.9	10.4	10.7	14.1	4.3	4.3	3.2	3.6	− 0.01	− 0.02
	AIc	6.3	6.0	7.3	8.8	8.6	8.6	2.5	2.5	1.4	4.0	0.01	− 0.07
	AIt	6.7	6.4	7.5	8.7	8.5	8.5	2.0	2.1	0.9	7.6	0.02	− 0.07
7 M	A1	0.0	0.0	0.0	0.0	0.0	0.0	0.0	0.0	0.0	6.8	− 0.05	− 0.07
	A3	5.3	5.0	5.8	3.0	2.4	4.2	− 2.3	− 2.7	− 1.6	6.8	− 0.04	− 0.02
	AEc	4.9	5.3	7.5	8.4	8.7	10.5	3.5	3.4	3.0	7.9	− 0.05	0.00
	AEt	11.8	12.2	16.1	12.9	13.2	16.1	1.0	1.0	0.0	7.1	− 0.02	− 0.02
	AIc	6.4	7.2	7.7	9.7	10.4	10.6	3.4	3.2	2.9	2.8	− 0.02	− 0.04
	AIt	6.3	6.1	6.3	9.4	9.2	9.1	3.1	3.1	2.8	1.5	− 0.02	− 0.05

* These values were obtained for the molecule at the stationary point with a single negative frequency equal to − 3.1 cm^−1^. The optimization could not converge to a minimum

Contreras and Madariaga also predict A19 as the most stable tautomer in the gas phase but point to A17 and AEc9 as second and third, respectively (Contreras and Madariaga 1998). They do not consider the A18 tautomer in their calculations, which were performed with the MP2/6-311++G(d,p) method. Solvation free energies obtained for three investigated tautomers are almost the same—within 1 kcal/mol. Our results show a much bigger variation of relative solvation free energies for these tautomers. The solvation free energies of A17 and AEc9 tautomers are around 2 kcal and 7 kcal/mol higher than those of A19, respectively. Their results point to the tautomer A17 as the most populated one in water solution. There are several possible reasons of these discrepancies—different solvation models (IPCM vs. IEFPCM in our study), calculation of solvation free energies on the gas phase optimal geometries used in ref. (Contreras and Madariaga 1998), and finally, different methods and basis sets. Nevertheless, comparison of the absorption spectra of natural 8AG (λ_max_ = 249 nm) and its methyl derivatives (λ_max_ = 251 nm for 9-methylated 8AG) clearly points to the tautomer protonated at position 9 as the most populated one (Wierzchowski et al. 2014), and therefore it rather supports our results. It should be noted that this interpretation is based on the assumption that methylation of the molecule at the most probable position of protonation does not significantly change the absorption spectra. A comparison of the vertical excitation energies of the natural and methylated forms of 8AG shown in Table 3 clearly supports the validity of this assumption.

**Table 3:** Vertical excitation and emission energies (eV) of selected low-energy tautomers and their methylated forms in water solution. All calculations were performed with the aug-cc-pvdz basis set. The oscillator strengths are provided in parentheses. The values best-matching the experimental data are marked in the boldface type. The Mean Absolute Error (MAE) is reported in the last line. All absorption energies are provided in SI. The original computed values are corrected by the expected systematic error of − 0.1 eV for excitation and 0.1 eV for emission (Avila Ferrer et al. 2013; Bai et al. 2020)

Compound		Excitation energy ${E}^{vert-a}$ (eV)						Emission energy ${E}^{vert-f}$ (eV)			
		B3LYP^b^		M06^b^	PBE0^b^		Exp^a^	B3LYP^c^	M06^c^	PBE0^c^	Exp^a^
NATURAL	A17	4.08 (0.11)		4.23 (0.12)	4.28 (0.12)		4.98	**3.00**	3.25	3.27	3.14
	A18	4.14 (0.11)		4.44 (0.12)	4.34 (0.12)			2.72	**3.07**	**3.04**	
	A19	4.60 (0.16)	**4.92**	4.79 (0.17)	4.80 (0.16)	5.07		3.45	3.74	3.71	
		5.04 (0.24)			5.23 (0.27)						
ANION	A1	**4.48 (0.12)**		4.63 (0.13)	4.66 (0.14)		4.46	**3.46**	2.83	**3.65**	3.49
	A3	4.77 (0.26)		4.91 (0.26)	4.95 (0.28)			1.16	**2.84**	4.02	
	A (da)	**4.49 (0.19)**		4.64 (0.18)	4.68 (0.21)		4.43	2.32	**3.32**	2.80	3.44
METHYL	A1m7	**4.10 (0.11)**		**4.24 (0.12)**	4.30 (0.12)		4.17	3.04	3.24	**3.27**	3.18
	A1m8	**4.16 (0.15)**		4.32 (0.17)	4.36 (0.17)		4.23	2.87	**3.18**	3.15	3.10
	A1m9	4.54 (0.17)	**4.86**	4.74 (0.20)	4.74 (0.18)	5.07	4.94	3.45	3.71	**3.69**	3.54
		5.02 (0.21)			5.20 (0.23)						
MET. ANIONS	AOam7 (ma)	**4.37 (0.25)**		4.28 (0.25)	4.59 (0.27)		4.48	**3.58**	2.82	3.80	3.43
	AOam8 (ma)	**4.04 (0.19)**		4.16 (0.21)	4.24 (0.21)		4.17	**2.95**	3.22	3.19	3.02
	AOam7 (ma)	**4.08 (0.12)**		4.19 (0.12)	4.28 (0.13)		4.13	2.86	**3.12**	3.14	3.06
NUCLEOSIDE		4.78 (0.23)	5.00	nc	4.78 (0.23)	4.96	nd	3.69	nc	3.69	nd
		5.21 (0.23)			5.14 (0.23)						
MAE (eV)		0.07		0.13	0.14			0.24	0.22	0.20	

*nc* not converged, *nd* no data, *ma* monoanion, *da* dianion

^a^The positions of maxima of experimental UV–Vis and fluorescence spectra of 8AG derivatives are taken from ref. (Wierzchowski et al. 2014)

^b^Original values are shifted by the expected systematic error of − 0.1 eV

^c^Original values are shifted by the expected systematic error of 0.1 eV

Tautomer protonated at position 1 dominates for all methylated neutral forms, as can be seen in Table 2, although this dominance is not so profound for molecules methylated in positions 7 and 8, for which A3 forms can be as close as ~ 3 kcal/mol comparing to ~ 9 kcal/mol for the molecule methylated at position 9. It seems that the large spatial separation of the tautomeric proton and the methyl group lowers the internal energy of the molecule. Replacement of a proton at position 9 by a methyl group significantly influences the Gibbs free energy balance of tautomers as the separation between tautomer protonated at position 1 and others significantly increases. The reverse effect was observed for 8aza-isoguanine, for which methylation at position 9 caused significant stabilization of amino-enol forms in water solution (Pyrka and Maciejczyk 2016). Also, the A3 tautomer of M9 and M8 methylated molecules has a significantly bigger dipole moment than the A1 form, and therefore the free energy of solvation is lower. It should be noted that the methyl group at position 9 mimics the presence of the sugar moiety in natural nucleosides, and strong discrimination of all non-A1 tautomeric forms is desired in the context of potential unwanted genomic mutations.

The Gibbs free energies of four tautomeric forms of monoanions—two amino-oxo forms A1 and A3 and two amino-enol forms AEc6 and AEt6—were calculated. Again, the dominant form is protonated at position 1, followed by the A3 tautomer with a free energy of 3.7 kcal/mol. Two amino-enol forms, AEc6 and AEt6, have free energies of 8.2 kcal/mol and 9.1 kcal/mol, respectively. Therefore, one can conclude that monoanionic forms are also dominated by tautomer protonated at position 1. Calculations for monoanions were performed only with the BHandHLYP/aug-cc-pVDZ/IEF-PCM methodology.

Extension of the basis set from aug-cc-pvdz to aug-cc-pvtz does not influence the energetical order of tautomers, but only affects relative Gibbs free energies, which are on average only slightly (~ 0.2 kcal/mol) higher for the larger basis set. The same conclusion pertains to the G3 composite method, for which relative free energies are on average significantly (~ 1.8 kcal/mol) higher than those obtained with the BHandHLYP/aug-cc-pvdz method, but the order of tautomers practically does not change.

### Zwitterions

In our previous study (Pyrka and Maciejczyk 2016), the possibility of the presence of zwitterionic species was checked for 8-aza-isoguanine methylated at position 9. A special test for the zwitterionic character of a molecule was developed, which went beyond the simple considerations of covalent bond orders. The total charges Q_P_ (pyrimidine ring) and Q_T_ (triazole ring) were computed, and a molecule was considered a zwitterion if the absolute values of both quantities is higher than 0.3 a.u. (for details, see ref. (Pyrka and Maciejczyk 2016)). It was shown that such analysis points to many more zwitterions than simple covalent bond order analysis. Therefore, the same procedure was applied to 8AG, and the results can be found in Tables 1 and 2. The distribution of charges between two rings is uniform, and no zwitterionic forms were detected, although it should be pointed out that zwitterionic species for methylated 8-aza-isoguanine were detected only for imino-enol forms and tautomers with proton and methyl groups located on the triazole ring (see Table 4 in Ref. (Pyrka and Maciejczyk 2016)). Additional calculations performed for 8AG for selected imino-enol forms and amino-oxo forms methylated at position 9 and with a tautomeric proton located on the triazole ring showed that zwitterionic charge distribution is also possible for some tautomers of 8AG (data not shown).

### Prediction of absorption and fluorescence peaks

The vertical excitation energies and the corresponding oscillator strengths of 8AG and its methylated and/or ionic forms are shown in Table 3. Almost all considered tautomers can be characterized by a single transition with oscillator strength significantly higher than zero in the vicinity of the maximum of the UV–Vis spectra. In such cases, only the single vertical transition energy is reported and compared to the experimental data. In some cases (e.g., A1m9), one can observe two vertical transitions with significant oscillator strengths, and for them, the energy of the transition was approximated by the position of the maximum of the curve, which was a superposition of two Gaussians with a half-width of 0.333 eV and maxima located at positions of vertical transition energies. The first five vertical excitation energies for all tautomers are shown in Tables S2–S5 of SI. All vertical excitation energies presented in Table 3 are corrected by the expected systematic error (Avila Ferrer et al. 2013) of − 0.1 eV, which is the mean value of the shift determined in the benchmark studies performed by (Bai et al. 2020). The authors of the latter publication proved that the origin of this systematic error is rooted in the frequency change between the ground state and excited states in multidimensional systems, and therefore vertical excitation and emission energies are expected to be blue- and red-shifted with respect to the positions of absorption and fluorescence peaks, respectively. The original (non-shifted) values of vertical transition energies are shown in Table S6 of SI.

The free energy difference between the most populated A19 tautomer and the second A18 tautomer is at least 3 kcal/mol, and therefore the absorption spectrum of this compound should practically reflect the absorption of the A19 tautomer. For all three tested methods, the best agreement between vertical absorption transitions and the maximum of the absorption spectra is obtained for the A19 tautomer. This is the case as well for A1 and A3 monoanions for which better agreement is obtained for the dominant A1 tautomer, for which free energy is 3.7 kcal/mol lower than A3 (BHandHLYP/aug-cc-pvdz).

Overall agreement between calculated vertical absorption transition energies and the experimental data is excellent for B3LYP functional, with mean absolute error (MAE) as low as 0.07 eV (uncorrected value 0.05 eV), which is significantly lower than 0.26 eV obtained in the benchmark of 60 excited valence states performed with TDDFT and B3LYP functional (Leang et al. 2012) or a similar value (0.27 eV) obtained for singlet excitations in the Thiel et al. benchmark set of 28 molecules (Schreiber et al. 2008; Laurent and Jacquemin 2013). Two other tested functionals performed significantly worse than B3LYP, with MAE = 0.13 eV (uncorrected value 0.17 eV) and 0.14 eV (uncorrected value 0.24 eV) for M06 and PBE0 functionals, respectively. Nevertheless, it should be noted that these values are still significantly better than the respective values obtained in the above-mentioned benchmarks, with 0.25 eV (M06) and 0.30 eV (PBE0) for the former and 0.28 eV (M06) and 0.24 eV (PBE0) for the later functional. It should also be noted that the average absolute difference of vertical excitation energies between forms methylated and protonated at the same positions equals only 0.02 eV for B3LYP functional and therefore supports the assumption of invariance of absorption spectra on proton → methyl substitution. It is important because the interpretation of experimental data is based on this assumption.

**Fig. 3:**
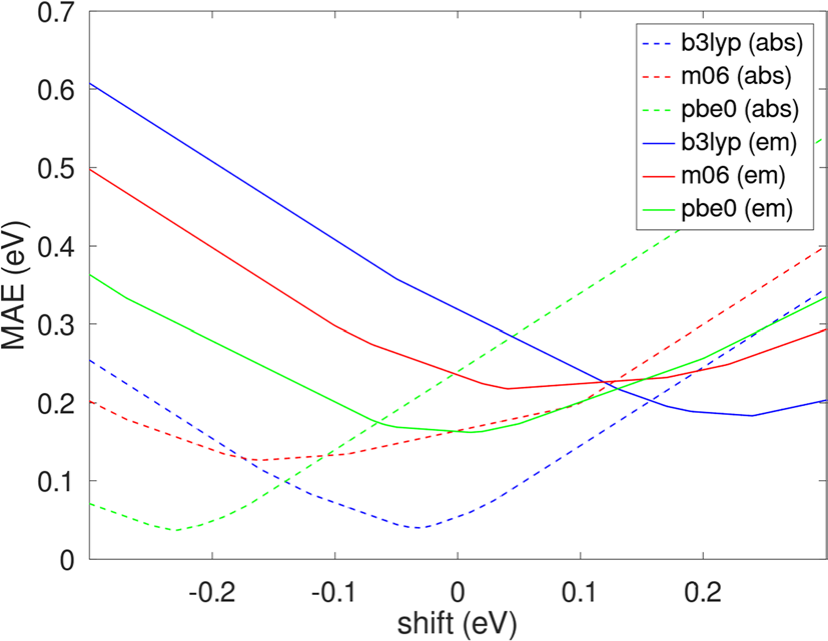
Mean absolute error (MAE) as a function of the shift of the computed vertical transition energies. MAEs for absorption and for emission are marked with dashed and solid lines, respectively

The vertical emission energies calculated for the investigated molecules are shown on the right-hand side of Table 3. These values were also corrected by the systematic error, which is expected to be in the range between 0.1 and 0.3 eV (Avila Ferrer et al. 2013). The values presented in Table 3 are corrected by 0.1 eV, the same (absolute) value which was used for vertical emission energies, but it should be noted that larger values of correction might lead to better agreement with experimental data in some cases (e.g., for neutral tautomers). The agreement between computed values and the experimental data, of course, depends on the assumed value of correction. The dependence of MAE on the value of shift for the three investigated functionals is shown in Fig. 3.

The raw data obtained for neutral non-methylated bases shows that the best agreement with the experimental data is achieved for the A17 tautomer using the M06 method (0.01 eV), although the PBE0 method is also very close to the experimental value of ${\lambda }_{max}$ (0.03 eV). Application of a 0.1 eV correction increases these differences and moves the value obtained with B3LYP significantly closer to the experimental value (0.14 eV). The interpretation of experimental spectra for non-methylated 8AG is based on a comparison of the positions of fluorescence peaks of natural and methylated forms. It is assumed that replacing the proton with the methyl group has a minor influence on the position of the maximum of the fluorescence spectra. The maximum of fluorescence spectra for neutral 8AG equals 395 nm and 350 nm, 400 nm, and 390 nm for 8AG methylated at positions 9, 8, and 7, respectively (Wierzchowski et al. 2014). Therefore, experimental data suggest that either the A18 or A17 tautomer (or both) is responsible for fluorescence. The raw results of TDDFT computations point to the A17 tautomer for which almost perfect agreement with the fluorescence spectra was achieved with PBE0 and M06 functionals, but inclusion of the expected systematic error leads to significant improvements of the agreement between A18 emission energy and experimental data also for M06 and PBE0 functionals. Therefore, the presented data cannot provide a definite answer to the question which of two tautomers is responsible for fluorescence.

For monoanions, the best agreement is obtained for 8AG protonated at position 1 using the B3LYP functional (difference of 0.03 eV for corrected value). The difference of 0.16 eV obtained with PBE0 is also good, but the M06 method completely missed the fluorescence peak for monoanions with a big energy difference of 0.67 eV. It should also be noted that our data points to the A1 anionic form as the one responsible for fluorescence. This is also a dominant form in water solution. For dianionic form, the best agreement was achieved with the M06 method, which underestimates vertical emission energy by 0.12 eV (with a 0.1 eV correction). Two other methods underestimate the emission energy by more than 0.6 eV.

For three methylated neutral forms protonated at position 1, very good agreement was obtained with the M06 and PBE0 methods with MAE = 0.1 eV for each of them, but the results obtained with B3LYP is also acceptable with MAE = 0.15 eV. It should be stressed that assessment of the performance of different functionals strongly depends on the value of systematic error correction, and increasing this value to 0.3 eV would lead to very good agreement of energies obtained with B3LYP and worsen the performance of M06 and PBE0 functionals. Also, the agreement with experimental data for methylated monoanions is very good with the following MAEs: 0.14 eV, 0.29 eV, and 0.21 eV for B3LYP, M06, and PBE0 methods, respectively. The relatively high MAE obtained with the M06 method is caused by the large error obtained for monoanion methylated at position 9. Overall, the best agreement of vertical emission energies was achieved with the PBE0 functional, which completely missed only the dianionic form (by 0.64 eV). Exclusion of the emission energy of dianion from MAE gives the value 0.14 eV, which should be considered a very good agreement with the experimental data.

As the vertical excitation and the emission energies are expected to be blue- and red-shifted, respectively (Avila Ferrer et al. 2013), the influence of the value of shift on MAE was determined, and it is shown in Fig. 3. The minimum of each curve corresponds to the optimal value of the shift for each method. Indeed, the presented results confirm that better agreement with experimental data is achieved with negative values of the shift for vertical excitation energies and positive ones for emission, although optimal values of the shift vary between different methods. A relatively large shift (~ − 0.2 eV) is expected for vertical excitations obtained with the M06 and PBE0 methods and for vertical emission obtained with the B3LYP method (~ + 0.25 eV). Interestingly, the order of optimal shift values for all methods is B3LYP > M06 > PBE0 for both vertical excitation and emission energies. The values of vertical transition energies corrected by optimal shift values are presented in Table S7. It can be seen that incorporation of optimal shifts makes the performance of prediction of absorption peaks by B3LYP and PBE0 practically indistinguishable with MAE = 0.04 eV for both methods and results in a slightly higher MAE for M06 (0.13 eV). Also, the performance of prediction of fluorescence peaks is improved, but MAEs for different methods are close to each other and vary from 0.16 eV (PBE0) via 0.19 eV (B3LYP) to 0.22 eV (M06). As the values of systematic errors resulting from the frequency change between ground and excited states (Bai et al. 2020) for all methods are not known, it is difficult to assess their performance. In this context, it seems that B3LYP and PBE0 perform slightly better than M06 for absorption, and PBE0 performs slightly better than the other two methods for emission, although the application of more sophisticated techniques like the nuclear ensemble approach (Crespo-Otero and Barbatti 2012) used in (Bai et al. 2020) seems to be necessary to provide a definite answer.

Additionally, vertical excitation and emission energies were computed for the 8AG nucleoside (previous to the last row in Table 3). The optimization of geometry did not converge for the M06 functional. For B3LYP and PBE0, the vertical excitation energies are 5.10 and 5.06 eV, respectively. These energies are around 0.1 eV larger than the experimental values obtained for 8AG and 9 m-8AG, but there is no experimental data available for the 8AG nucleoside. The vertical emission energies obtained with B3LYP and PBE0 functionals are almost identical (3.59 eV) and equal to the emission energy computed with PBE0 for the A1m9 molecule. This value is also only 0.05 eV larger than the experimental value measured for 9 m-8AG. Therefore, one can conclude that absorption spectra should not be affected by methylation of 8AG at position 9, but can be slightly shifted by ribosylation at the same position. Our results also suggest that the fluorescence spectra for 8AG methylated and ribosylated at position 9 should be very similar.

**Fig. 4:**
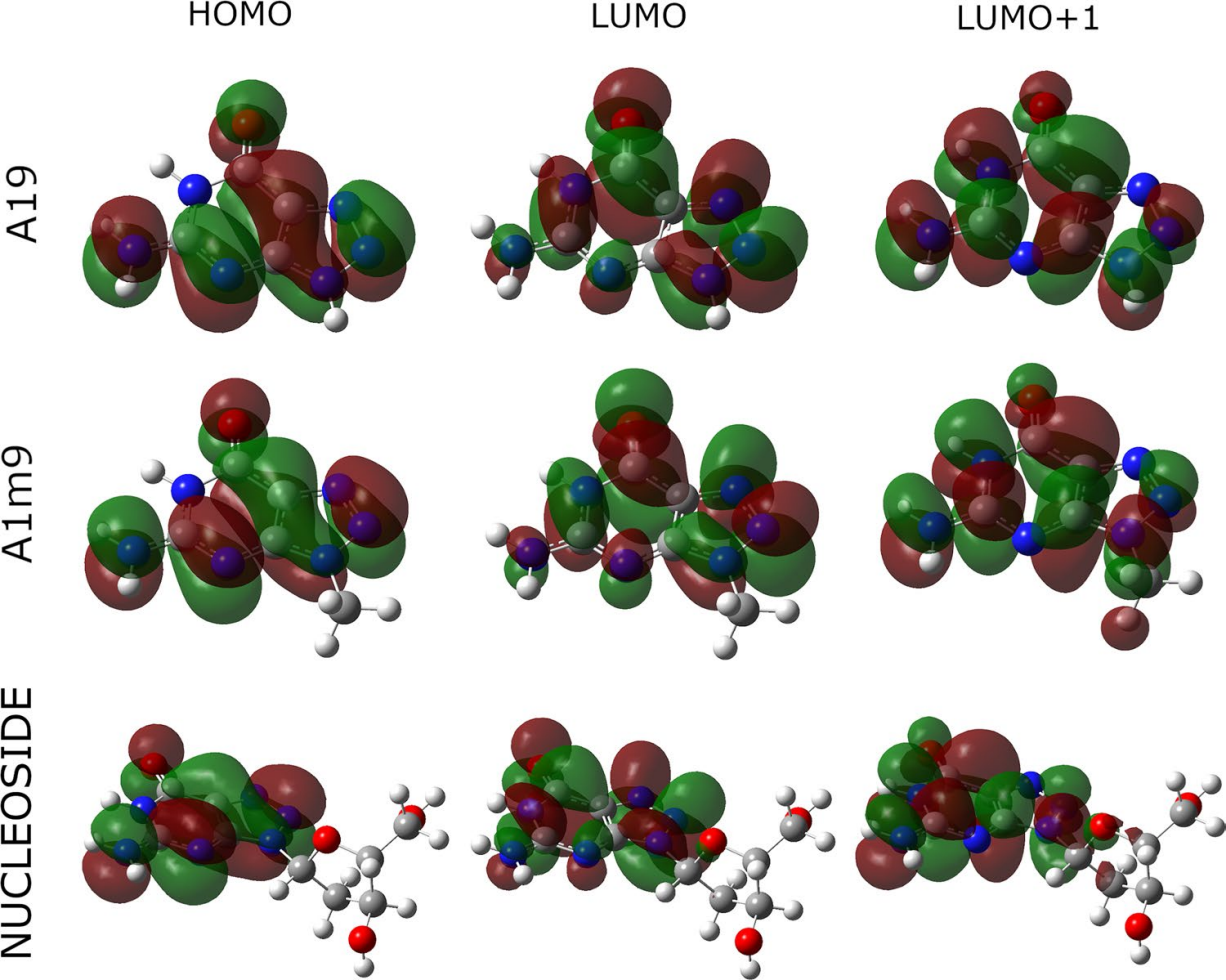
Comparison of molecular orbitals involved in S1 (HOMO → LUMO) and S2 (HOMO → LUMO + 1) transitions for A19, A1m9, and 8AG nucleoside. All calculations were performed at the B3LYP/aug-cc-pvdz level of theory in an IEF-PCM-modeled water solution. The figure was prepared with GaussView

Molecular orbitals involved in S1 and S2 transitions for A19, A1m9, and 8AG nucleosides are shown in Fig. 4. For all molecules, the S1 state arises mainly from HOMO → LUMO transition and the S2 state from HOMO → LUMO+1. As expected, the orbitals involved in vertical excitations of the above-mentioned molecules are qualitatively very similar, and only a negligible amount of wavefunction is localized on the methyl group of the LUMO+1 orbital of A1m9 and the sugar ring of LUMO+1 of 8AG nucleoside. Both transitions have a ππ^*^ character.

## Conclusions

In this publication, the tautomeric equilibrium of 8-azaguanine and its methylated derivatives was determined in the gas phase and in water solution using three methods: hybrid BHandHLYP with aug-cc-pvdz and aug-cc-pvtz basis sets, and composite G3. All methods point to the A19 tautomer as the one most populated in the gas phase, followed by also significantly populated A18, AEc9, and AEt9 forms. In water, A19 is the sole dominant tautomeric form (> 99%), in line with the interpretation of the absorption spectra of natural 8AG and its methylated forms. Also, the tautomer protonated at position 1 is the most populated one for all methyl derivatives of 8AG, although amino-enol tautomers AEc and AEt of 9-methylated 8AG cannot be neglected in the gas phase. Methylation of 8AG at position 9 causes a large stabilization of the A1 form with respect to other tautomers. Extension of the basis set to aug-cc-pvtz does not influence the order of tautomers, but relative free energies are on average slightly higher with the extended basis set. The results obtained with the hybrid G3 method are also qualitatively very similar.

Vertical excitation and emission energies were calculated with the three functionals B3LYP, M06, and PBE0 combined with the aug-cc-pvdz basis set. Excellent agreement of the positions of absorption peaks was achieved for B3LYP, with MAE equal to 0.07 eV for all investigated compounds. The PBE0 functional seems to be the best choice for calculation of vertical emission energies with MAE = 0.20 eV, although exclusion of di-anion, which was completely overshoot by the method, lowers MAE to 0.14 eV. Comparison of vertical absorption energies of natural and methylated forms of 8AG supports the assumption of invariance of absorption spectra on proton → methyl replacement, which is crucial for interpretation of the experimental data.

Finally, based on the experimental data, it is not possible to choose whether A17 or A18 is the tautomer responsible for the fluorescence spectra of 8AG. Although the best match of vertical emission energies with experimental data was obtained for A17 tautomer (raw data), inclusion of the expected (positive) systematic error improves the agreement of experimental data with vertical emission energies for A18 tautomer for M06 and PBE0 methods. Therefore, obtained results do not provide a definite answer to this question. Also, the good agreement of vertical emission energies for methylated 8AG with fluorescence peaks combined with their lowest free energy strongly suggests that tautomers protonated at position 1 are responsible for emission the spectra of methylated molecules.

### Supplementary Information

Below is the link to the electronic supplementary material.

Supplementary file1 (DOCX 61 KB)

## Data Availability

The data that support the findings of this study are available from the corresponding author on request.
