# Supplementary material for: Tautomeric equilibrium and spectroscopic properties of 8-azaguanine revealed by quantum chemistry methods
Source: Eur Biophys J. 2023 Jul 28;52(6-7):545–57. doi: 10.1007/s00249-023-01672-x (PMC10618388; doi:10.1007/s00249-023-01672-x)
Supplement: Supplementary file 1 — Supplementary file1 (DOCX 61 KB) [file 249_2023_1672_MOESM1_ESM.docx]

**Tautomeric equilibrium and spectroscopic properties of 8-azaguanine revealed by quantum chemistry methods**

Maciej Maciejczyk ^a,*^, Maciej Pyrka^a^

Supplementary Information

^a^ Department of Physics and Biophysics, University of Warmia and Mazury in Olsztyn, Olsztyn, Poland;

* Corresponding author: maciej.maciejczyk@uwm.edu.pl

**Table S1.** Decomposition of Gibbs free energies ΔG of 8AG tautomers and their methylated forms determined in the gas phase and in water. ΔE is ZPE-corrected relative energy, ΔH is a relative enthalpy, and ΔS is a relative entropy (T=300K). All values are provided in kcal/mol and computed with the BHandHLYP/aug-cc-pVDZ method. In each group, the reference is tautomer with all zero values. Populations of tautomers are determined from the Boltzmann distribution.

| Compound | Form | ΔE | ΔH | TΔS | ΔG | pop | ΔE | ΔH | TΔS | ΔG | pop |
| --- | --- | --- | --- | --- | --- | --- | --- | --- | --- | --- | --- |
|  |  | gas | | | | | water | | | | |
| 8AG | A17 | 2.48 | 2.41 | 0.12 | 2.5 | 9.3٠10^-3^ | 3.95 | 3.78 | -0.57 | 4.4 | 6.1٠10^-4^ |
|  | A18 | 0.74 | 0.64 | -0.17 | 0.8 | 0.16 | 2.55 | 2.37 | -0.59 | 3.0 | 6.4٠10^-3^ |
|  | A19 | 0.00 | 0.00 | 0.00 | 0.0 | 0.62 | 0.00 | 0.00 | 0.00 | 0.0 | 0.99 |
|  | A37 | 8.48 | 8.56 | 0.20 | 8.4 | 4.6٠10^-7^ | 7.61 | 7.68 | 0.15 | 7.5 | 3.3٠10^-6^ |
|  | A38 | 9.31 | 9.40 | 0.24 | 9.2 | 1.2٠10^-7^ | 6.81 | 6.76 | -0.24 | 7.0 | 7.7٠10^-6^ |
|  | A39 | 18.08 | 18.23 | 0.34 | 17.9 | 5.3٠10^-14^ | 9.09 | 9.12 | -0.05 | 9.2 | 1.9٠10^-7^ |
|  | AEc7 | 6.63 | 6.63 | -0.08 | 6.7 | 8.0٠10^-6^ | 11.95 | 11.76 | -0.67 | 12.4 | 8.8٠10^-10^ |
|  | AEt7 | 13.49 | 13.68 | 0.30 | 13.4 | 1.0٠10^-10^ | 15.24 | 15.07 | -0.65 | 15.7 | 3.4٠10^-12^ |
|  | AEc8 | 5.20 | 5.14 | -0.12 | 5.3 | 8.4٠10^-5^ | 11.63 | 11.44 | -0.62 | 12.1 | 1.5٠10^-9^ |
|  | AEt8 | 7.06 | 7.13 | 0.26 | 6.9 | 5.7٠10^-7^ | 13.24 | 13.08 | -0.56 | 13.6 | 1.2٠10^-10^ |
|  | AEc9 | 0.94 | 1.04 | 0.29 | 0.8 | 0.16 | 7.57 | 7.56 | -0.05 | 7.6 | 2.8٠10^-6^ |
|  | AEt9 | 1.93 | 2.10 | 0.61 | 1.5 | 0.05 | 8.99 | 9.01 | 0.03 | 9.0 | 2.7٠10^-7^ |
|  | AIc7 | 9.62 | 9.52 | 0.02 | 9.5 | 7.2٠10^-8^ | 14.11 | 13.82 | -0.63 | 14.5 | 2.6٠10^-11^ |
|  | AIt7 | 9.56 | 9.48 | 0.05 | 9.4 | 8.5٠10^-8^ | 13.89 | 13.61 | -0.62 | 14.2 | 4.3٠10^-11^ |
|  | AIc8 | 7.25 | 7.14 | -0.01 | 7.1 | 4.1٠10^-6^ | 11.64 | 11.33 | -0.69 | 12.0 | 1.7٠10^-9^ |
|  | AIt8 | 7.58 | 7.49 | 0.03 | 7.5 | 2.1٠10^-6^ | 11.45 | 11.12 | -0.73 | 11.9 | 2.0٠10^-9^ |
|  | AIc9 | 13.30 | 13.28 | 0.16 | 13.1 | 1.7٠10^-10^ | 12.70 | 12.45 | -0.55 | 13.0 | 3.2٠10^-10^ |
|  | AIt9 | 14.92 | 14.95 | 0.27 | 14.7 | 1.2٠10^-11^ | 12.82 | 12.58 | -0.58 | 13.2 | 2.3٠10^-10^ |
| 9m-8AG | A1m9 | 0.00 | 0.00 | 0.00 | 0.0 | 0.81 | 0.00 | 0.00 | 0.00 | 0.0 | 1.0 |
|  | A3m9* | 17.94 | 18.03 | 0.02 | 18.0 | 5.9٠10^-14^ | 9.08 | 8.57 | -1.82 | 10.4 | 2.6٠10^-8^ |
|  | AEcm9 | 1.11 | 1.16 | 0.11 | 1.1 | 0.13 | 7.72 | 7.61 | -0.70 | 8.3 | 8.7٠10^-7^ |
|  | AEtm9 | 1.93 | 2.12 | 0.61 | 1.5 | 0.06 | 9.12 | 9.04 | -0.59 | 9.6 | 9.8٠10^-8^ |
|  | AIcm9 | 12.93 | 12.86 | -0.14 | 13.0 | 2.6٠10^-10^ | 13.03 | 12.66 | -1.31 | 14.0 | 6.0٠10^-11^ |
|  | AItm9 | 14.55 | 14.53 | -0.04 | 14.6 | 1.8٠10^-11^ | 13.18 | 12.82 | -1.22 | 14.0 | 6.0٠10^-11^ |
| 8m-8AG | A1m8 | 0.00 | 0.00 | 0.00 | 0.0 | 0.999 | 0.00 | 0.00 | 0.00 | 0.0 | 0.998 |
|  | A3m8 | 8.84 | 9.02 | 0.49 | 8.5 | 6.2٠10^-7^ | 4.22 | 4.45 | 0.66 | 3.8 | 1.7٠10^-3^ |
|  | AEcm8 | 4.24 | 4.25 | -0.05 | 4.3 | 7.2٠10^-4^ | 8.78 | 8.78 | -0.02 | 8.8 | 3.8٠10^-7^ |
|  | AEtm8 | 6.21 | 6.33 | 0.25 | 6.1 | 3.5٠10^-5^ | 10.37 | 10.40 | 0.04 | 10.4 | 2.6٠10^-8^ |
|  | AIcm8 | 6.47 | 6.44 | 0.11 | 6.3 | 2.5٠10^-5^ | 8.93 | 8.85 | 0.04 | 8.8 | 3.8٠10^-7^ |
|  | AItm8 | 6.85 | 6.86 | 0.19 | 6.7 | 1.3٠10^-5^ | 8.79 | 8.69 | -0.03 | 8.7 | 4.6٠10^-7^ |
| 7m-8AG | A1m7 | 0.00 | 0.00 | 0.00 | 0.0 | 0.995 | 0.00 | 0.00 | 0.00 | 0.0 | 0.994 |
|  | A3m7 | 5.63 | 5.79 | 0.48 | 5.3 | 1.4٠10^-4^ | 3.60 | 3.86 | 0.87 | 3.0 | 6.4٠10^-3^ |
|  | AEcm7 | 4.72 | 4.73 | -0.18 | 4.9 | 2.7٠10^-4^ | 8.29 | 8.23 | -0.17 | 8.4 | 7.3٠10^-7^ |
|  | AEtm7 | 11.57 | 11.63 | -0.21 | 11.8 | 2.4٠10^-9^ | 12.46 | 12.29 | -0.60 | 12.9 | 3.8٠10^-10^ |
|  | AIcm7 | 6.54 | 6.50 | 0.05 | 6.4 | 2.1٠10^-5^ | 9.67 | 9.64 | -0.06 | 9.7 | 8.3٠10^-8^ |
|  | AItm7 | 6.42 | 6.40 | 0.09 | 6.3 | 2.5٠10^-5^ | 9.55 | 9.44 | 0.01 | 9.4 | 1.4٠10^-7^ |

* For this compound optimization could not find stationary point without negative frequencies. These vertical excitation energies were computed for stationary point with single (first) negative frequency equal -3.1 cm^-1^.

**Table S2**. First five vertical excitation energies of 8AG tautomers and their methylated forms. All calculations were performed on the B3LYP/aug-cc-pvdz level of theory in water solution modeled by the IEF-PCM model. All energies (the first number) are provided in eV, wavelength in nm in parentheses, and the final number is the oscillator strength.

| Form | S1 | S2 | S3 | S4 | S5 |
| --- | --- | --- | --- | --- | --- |
| 8AG | | | | | |
| A17 | 4.18 (297) 0.11 | 4.83 (256) 0.00 | 5.13 (242) 0.00 | 5.40 (230) 0.18 | 5.46 (227) 0.01 |
| A18 | 4.24 (292) 0.11 | 4.85 (256) 0.00 | 5.33 (233) 0.00 | 5.36 (231) 0.14 | 5.53 (224) 0.00 |
| A19 | 4.70 (264) 0.16 | 5.14 (241) 0.24 | 5.19 (239) 0.00 | 5.28 (235) 0.00 | 5.53 (224) 0.00 |
| A37 | 4.45 (279) 0.00 | 4.74 (262) 0.19 | 5.06 (245) 0.13 | 5.30 (234) 0.00 | 5.67 (219) 0.00 |
| A38 | 4.35 (285) 0.00 | 4.83 (257) 0.20 | 5.02 (247) 0.18 | 5.32 (233) 0.00 | 5.93 (209) 0.00 |
| A39 | 4.62 (268) 0.00 | 5.11 (243) 0.31 | 5.28 (235) 0.10 | 5.52 (225) 0.00 | 5.67 (219) 0.00 |
| AEc7 | 3.99 (311) 0.10 | 4.63 (268) 0.00 | 5.08 (244) 0.01 | 5.42 (229) 0.01 | 5.44 (228) 0.00 |
| AEt7 | 3.96 (313) 0.09 | 4.57 (272) 0.00 | 5.03 (247) 0.01 | 5.35 (232) 0.00 | 5.39 (230) 0.02 |
| AEc8 | 3.99 (311) 0.12 | 4.52 (275) 0.00 | 5.26 (236) 0.04 | 5.50 (226) 0.00 | 5.61 (221) 0.00 |
| AEt8 | 3.97 (313) 0.12 | 4.45 (279) 0.00 | 5.25 (236) 0.05 | 5.50 (225) 0.00 | 5.63 (220) 0.00 |
| AEc9 | 4.42 (281) 0.18 | 4.86 (255) 0.00 | 5.30 (234) 0.04 | 5.34 (232) 0.01 | 5.53 (224) 0.00 |
| AEt9 | 4.39 (282) 0.18 | 4.83 (257) 0.00 | 5.29 (234) 0.04 | 5.31 (234) 0.01 | 5.54 (223) 0.00 |
| AIc7 | 4.10 (302) 0.07 | 4.85 (256) 0.00 | 5.39 (230) 0.00 | 5.51 (225) 0.00 | 5.59 (222) 0.12 |
| AIt7 | 4.16 (298) 0.07 | 4.92 (252) 0.00 | 5.40 (229) 0.00 | 5.52 (224) 0.00 | 5.58 (222) 0.12 |
| AIc8 | 4.28 (290) 0.10 | 4.84 (256) 0.00 | 5.60 (221) 0.09 | 5.61 (221) 0.01 | 5.81 (213) 0.18 |
| AIt8 | 4.34 (285) 0.10 | 4.91 (252) 0.00 | 5.59 (222) 0.10 | 5.62 (221) 0.01 | 5.82 (213) 0.15 |
| AIc9 | 4.69 (264) 0.11 | 5.15 (241) 0.00 | 5.49 (226) 0.26 | 5.58 (222) 0.00 | 5.65 (220) 0.01 |
| AIt9 | 4.75 (261) 0.11 | 5.21 (238) 0.00 | 5.51 (225) 0.25 | 5.58 (222) 0.00 | 5.66 (219) 0.01 |
| 9m-8AG | | | | | |
| A1m9 | 4.64 (267) 0.17 | 5.12 (242) 0.21 | 5.18 (239) 0.00 | 5.25 (236) 0.01 | 5.46 (227) 0.00 |
| A3m9 | 4.63 (268) 0.00 | 5.08 (244) 0.32 | 5.28 (235) 0.09 | 5.51 (225) 0.00 | 5.67 (219) 0.00 |
| AEcm9 | 4.51 (275) 0.19 | 4.94 (251) 0.00 | 5.34 (232) 0.02 | 5.36 (231) 0.01 | 5.49 (226) 0.00 |
| AEtm9 | 4.48 (277) 0.19 | 4.92 (252) 0.00 | 5.32 (233) 0.01 | 5.33 (232) 0.02 | 5.50 (225) 0.00 |
| AIcm9 | 4.67 (266) 0.11 | 5.15 (241) 0.00 | 5.47 (227) 0.23 | 5.53 (224) 0.00 | 5.63 (220) 0.01 |
| AItm9 | 4.72 (263) 0.12 | 5.21 (238) 0.00 | 5.49 (226) 0.23 | 5.53 (224) 0.00 | 5.64 (220) 0.01 |
| 8m-8AG | | | | | |
| A1m8 | 4.26 (291) 0.15 | 4.90 (253) 0.00 | 5.28 (235) 0.16 | 5.39 (230) 0.00 | 5.44 (228) 0.01 |
| A3m8 | 4.40 (282) 0.00 | 4.77 (260) 0.30 | 5.05 (246) 0.17 | 5.37 (231) 0.00 | 5.84 (212) 0.00 |
| AEcm8 | 4.16 (300) 0.17 | 4.68 (265) 0.00 | 5.35 (232) 0.04 | 5.43 (228) 0.01 | 5.67 (219) 0.00 |
| AEtm8 | 4.14 (300) 0.16 | 4.61 (269) 0.00 | 5.34 (232) 0.04 | 5.43 (228) 0.01 | 5.69 (218) 0.00 |
| AIcm8 | 4.23 (293) 0.13 | 4.87 (254) 0.00 | 5.48 (226) 0.01 | 5.56 (223) 0.09 | 5.74 (216) 0.16 |
| AItm8 | 4.29 (289) 0.14 | 4.94 (251) 0.00 | 5.49 (226) 0.01 | 5.56 (223) 0.10 | 5.91 (210) 0.00 |
| 7m-8AG | | | | | |
| A1m7 | 4.20 (295) 0.11 | 4.89 (254) 0.00 | 5.15 (241) 0.00 | 5.35 (232) 0.16 | 5.40 (230) 0.01 |
| A3m7 | 4.49 (276) 0.00 | 4.76 (261) 0.18 | 5.12 (242) 0.14 | 5.35 (232) 0.00 | 5.69 (218) 0.00 |
| AEcm7 | 4.16 (298) 0.09 | 4.76 (261) 0.00 | 5.17 (240) 0.01 | 5.41 (229) 0.01 | 5.45 (227) 0.02 |
| AEtm7 | 4.13 (300) 0.09 | 4.69 (264) 0.00 | 5.10 (243) 0.01 | 5.40 (229) 0.01 | 5.44 (228) 0.03 |
| AIcm7 | 4.10 (302) 0.07 | 4.89 (253) 0.00 | 5.37 (231) 0.00 | 5.44 (228) 0.01 | 5.56 (223) 0.09 |
| AItm7 | 4.12 (295) 0.07 | 4.97 (249) 0.00 | 5.45 (227) 0.00 | 5.47 (227) 0.01 | 5.59 (222) 0.11 |

**Table S3**. The same as Table S2, but with M06 functional.

| Form | S1 | S2 | S3 | S4 | S5 |
| --- | --- | --- | --- | --- | --- |
| 8AG | | | | | |
| A17 | 4.33 (287) 0.12 | 4.87 (254) 0.00 | 4.95 (251) 0.00 | 5.20 (238) 0.00 | 5.27 (236) 0.01 |
| A18 | 4.54 (273) 0.12 | 5.03 (247) 0.00 | 5.08 (244) 0.00 | 5.38 (230) 0.00 | 5.49 (226) 0.00 |
| A19 | 4.82 (257) 0.18 | 4.96 (250) 0.00 | 5.17 (240) 0.26 | 5.18 (239) 0.01 | 5.26 (236) 0.00 |
| A37 | 4.50 (275) 0.00 | 4.82 (257) 0.20 | 5.27 (235) 0.14 | 5.30 (234) 0.01 | 5.38 (231) 0.00 |
| A38 | 4.41 (281) 0.00 | 4.94 (251) 0.22 | 5.22 (237) 0.19 | 5.39 (230) 0.00 | 5.52 (225) 0.00 |
| A39 | 4.69 (265) 0.00 | 5.20 (238) 0.32 | 5.35 (232) 0.00 | 5.46 (227) 0.07 | 5.48 (226) 0.01 |
| AEc7 | 4.08 (304) 0.11 | 4.60 (269) 0.00 | 4.95 (251) 0.00 | 5.03 (247) 0.01 | 5.25 (236) 0.00 |
| AEt7 | 4.06 (306) 0.11 | 4.55 (273) 0.00 | 4.89 (253) 0.00 | 4.96 (250) 0.01 | 5.20 (238) 0.00 |
| AEc8 | 4.11 (302) 0.13 | 4.52 (274) 0.00 | 5.02 (247) 0.00 | 5.32 (233) 0.01 | 5.34 (232) 0.03 |
| AEt8 | 4.09 (303) 0.13 | 4.45 (279) 0.00 | 5.02 (247) 0.00 | 5.30 (234) 0.01 | 5.32 (233) 0.04 |
| AEc9 | 4.53 (273) 0.21 | 4.85 (256) 0.00 | 5.04 (246) 0.00 | 5.30 (234) 0.01 | 5.37 (231) 0.05 |
| AEt9 | 4.51 (275) 0.21 | 4.82 (257) 0.00 | 5.05 (245) 0.00 | 5.25 (236) 0.01 | 5.36 (231) 0.05 |
| AIc7 | 4.27 (291) 0.08 | 4.92 (252) 0.00 | 5.03 (247) 0.00 | 5.34 (232) 0.00 | 5.42 (229) 0.00 |
| AIt7 | 4.32 (287) 0.09 | 4.99 (248) 0.00 | 5.04 (246) 0.00 | 5.36 (231) 0.00 | 5.42 (229) 0.00 |
| AIc8 | 4.47 (277) 0.11 | 4.92 (252) 0.00 | 5.14 (241) 0.00 | 5.50 (225) 0.00 | 5.67 (219) 0.00 |
| AIt8 | 4.53 (273) 0.12 | 4.99 (249) 0.00 | 5.15 (241) 0.00 | 5.58 (222) 0.00 | 5.71 (217) 0.00 |
| AIc9 | 4.88 (254) 0.13 | 5.09 (244) 0.00 | 5.22 (237) 0.00 | 5.51 (225) 0.00 | 5.57 (223) 0.01 |
| AIt9 | 4.93 (252) 0.13 | 5.08 (244) 0.00 | 5.28 (235) 0.00 | 5.59 (222) 0.01 | 5.63 (220) 0.00 |
| 9m-8AG | | | | | |
| A1m9 | 4.84 (256) 0.20 | 5.30 (234) 0.00 | 5.34 (232) 0.01 | 5.40 (230) 0.00 | 5.72 (217) 0.00 |
| A3m9* | 4.74 (262) 0.00 | 5.22 (238) 0.32 | 5.33 (233) 0.00 | 5.50 (226) 0.07 | 5.50 (226) 0.01 |
| AEcm9 | 4.63 (268) 0.22 | 4.92 (252) 0.00 | 5.01 (248) 0.00 | 5.33 (233) 0.01 | 5.39 (230) 0.02 |
| AEtm9 | 4.60 (269) 0.21 | 4.90 (253) 0.00 | 5.02 (247) 0.00 | 5.27 (235) 0.01 | 5.35 (232) 0.00 |
| AIcm9 | 4.85 (256) 0.14 | 5.08 (244) 0.00 | 5.22 (238) 0.00 | 5.52 (225) 0.00 | 5.56 (223) 0.01 |
| AItm9 | 4.90 (253) 0.14 | 5.08 (244) 0.00 | 5.27 (235) 0.00 | 5.58 (222) 0.01 | 5.62 (221) 0.00 |
| 8m-8AG | | | | | |
| A1m8 | 4.42 (280) 0.17 | 4.95 (250) 0.00 | 5.02 (247) 0.00 | 5.29 (234) 0.01 | 5.39 (230) 0.16 |
| A3m8 | 4.40 (282) 0.00 | 4.77 (260) 0.30 | 5.05 (246) 0.17 | 5.37 (231) 0.00 | 5.84 (212) 0.00 |
| AEcm8 | 4.27 (290) 0.19 | 4.68 (265) 0.00 | 4.96 (250) 0.00 | 5.30 (234) 0.00 | 5.41 (230) 0.03 |
| AEtm8 | 4.24 (292) 0.19 | 4.61 (269) 0.00 | 4.95 (250) 0.00 | 5.26 (236) 0.00 | 5.40 (230) 0.04 |
| AIcm8 | 4.40 (282) 0.16 | 4.94 (251) 0.00 | 5.00 (248) 0.01 | 5.41 (229) 0.00 | 5.54 (224) 0.00 |
| AItm8 | 4.46 (278) 0.17 | 5.01 (248) 0.00 | 5.01 (247) 0.01 | 5.47 (227) 0.00 | 5.58 (222) 0.00 |
| 7m-8AG | | | | | |
| A1m7 | 4.34 (286) 0.12 | 4.90 (253) 0.00 | 4.93 (252) 0.00 | 5.21 (238) 0.00 | 5.28 (235) 0.00 |
| A3m7 | 4.59 (270) 0.00 | 4.88 (254) 0.18 | 5.29 (234) 0.00 | 5.37 (231) 0.15 | 5.37 (231) 0.00 |
| AEcm7 | 4.25 (292) 0.11 | 4.72 (262) 0.00 | 4.93 (251) 0.00 | 5.13 (242) 0.01 | 5.28 (235) 0.00 |
| AEtm7 | 4.22 (294) 0.10 | 4.67 (266) 0.00 | 4.96 (250) 0.00 | 5.05 (245) 0.01 | 5.26 (236) 0.00 |
| AIcm7 | 4.26 (291) 0.08 | 4.96 (250) 0.00 | 4.97 (250) 0.00 | 5.31 (234) 0.00 | 5.42 (229) 0.00 |
| AItm7 | 4.35 (284) 0.08 | 4.99 (248) 0.00 | 5.03 (246) 0.00 | 5.40 (230) 0.00 | 5.43 (228) 0.00 |

* For this compound optimization could not find stationary point without negative frequencies. These vertical excitation energies were computed for stationary point with single (first) negative eigenvalue equal -19.0 cm^-1^.

**Table S4**. The same as Table S2 but with PBE0 functional.

| Form | S1 | S2 | S3 | S4 | S5 |
| --- | --- | --- | --- | --- | --- |
| 8AG | | | | | |
| A17 | 4.38 (283) 0.12 | 5.01 (248) 0.00 | 5.32 (233) 0.00 | 5.59 (222) 0.18 | 5.72 (217) 0.01 |
| A18 | 4.44 (279) 0.12 | 5.02 (247) 0.00 | 5.56 (223) 0.00 | 5.59 (222) 0.14 | 5.76 (215) 0.00 |
| A19 | 4.70 (264) 0.16 | 5.14 (241) 0.24 | 5.19 (239) 0.00 | 5.28 (235) 0.00 | 5.53 (224) 0.00 |
| A37 | 4.45 (279) 0.00 | 4.74 (262) 0.19 | 5.06 (245) 0.13 | 5.30 (234) 0.00 | 5.67 (219) 0.00 |
| A38 | 4.35 (285) 0.00 | 4.83 (257) 0.20 | 5.02 (247) 0.18 | 5.32 (233) 0.00 | 5.94 (209) 0.00 |
| A39 | 4.63 (268) 0.00 | 5.11 (243) 0.31 | 5.28 (235) 0.10 | 5.52 (225) 0.00 | 5.67 (219) 0.00 |
| AEc7 | 3.99 (311) 0.10 | 4.63 (268) 0.00 | 5.08 (244) 0.01 | 5.42 (229) 0.01 | 5.44 (228) 0.00 |
| AEt7 | 3.96 (313) 0.09 | 4.57 (272) 0.00 | 5.03 (247) 0.01 | 5.35 (232) 0.00 | 5.39 (230) 0.02 |
| AEc8 | 3.99 (311) 0.12 | 4.52 (275) 0.00 | 5.26 (236) 0.04 | 5.50 (226) 0.00 | 5.61 (221) 0.00 |
| AEt8 | 3.97 (313) 0.12 | 4.45 (279) 0.00 | 5.25 (236) 0.05 | 5.50 (225) 0.00 | 5.63 (220) 0.00 |
| AEc9 | 4.42 (281) 0.18 | 4.86 (255) 0.00 | 5.30 (234) 0.04 | 5.34 (232) 0.01 | 5.53 (224) 0.00 |
| AEt9 | 4.39 (282) 0.18 | 4.83 (257) 0.00 | 5.29 (234) 0.04 | 5.31 (234) 0.01 | 5.54 (224) 0.00 |
| AIc7 | 4.10 (302) 0.07 | 4.85 (256) 0.00 | 5.39 (230) 0.00 | 5.51 (225) 0.00 | 5.59 (222) 0.12 |
| AIt7 | 4.16 (298) 0.07 | 4.92 (252) 0.00 | 5.40 (229) 0.00 | 5.52 (224) 0.00 | 5.58 (222) 0.12 |
| AIc8 | 4.28 (290) 0.10 | 4.84 (256) 0.00 | 5.60 (221) 0.09 | 5.61 (221) 0.01 | 5.81 (213) 0.18 |
| AIt8 | 4.34 (285) 0.10 | 4.91 (252) 0.00 | 5.59 (222) 0.10 | 5.62 (221) 0.01 | 5.82 (213) 0.15 |
| AIc9 | 4.69 (264) 0.11 | 5.15 (241) 0.00 | 5.49 (226) 0.26 | 5.58 (222) 0.00 | 5.65 (220) 0.01 |
| AIt9 | 4.75 (261) 0.11 | 5.21 (238) 0.00 | 5.51 (225) 0.25 | 5.58 (222) 0.00 | 5.66 (219) 0.01 |
| 9m-8AG | | | | | |
| A1m9 | 4.84 (256) 0.18 | 5.30 (234) 0.23 | 5.34 (232) 0.00 | 5.40 (230) 0.01 | 5.71 (217) 0.00 |
| A3m9 | 4.78 (259) 0.00 | 5.28 (235) 0.34 | 5.51 (225) 0.09 | 5.69 (218) 0.01 | 5.88 (211) 0.00 |
| AEcm9 | 4.65 (267) 0.20 | 5.07 (244) 0.00 | 5.47 (227) 0.02 | 5.48 (226) 0.01 | 5.74 (216) 0.00 |
| AEtm9 | 4.62 (268) 0.20 | 5.05 (245) 0.00 | 5.44 (228) 0.01 | 5.47 (227) 0.02 | 5.75 (216) 0.00 |
| AIcm9 | 4.83 (257) 0.12 | 5.25 (236) 0.00 | 5.63 (220) 0.25 | 5.75 (216) 0.01 | 5.78 (214) 0.00 |
| AItm9 | 4.88 (254) 0.13 | 5.30 (234) 0.00 | 5.65 (219) 0.25 | 5.77 (215) 0.00 | 5.78 (215) 0.01 |
| 8m-8AG | | | | | |
| A1m8 | 4.46 (278) 0.17 | 5.06 (245) 0.00 | 5.46 (227) 0.18 | 5.63 (220) 0.00 | 5.69 (218) 0.01 |
| A3m8 | 4.57 (271) 0.00 | 4.96 (250) 0.33 | 5.30 (234) 0.19 | 5.62 (220) 0.00 | 6.08 (204) 0.00 |
| AEcm8 | 4.30 (289) 0.18 | 4.82 (257) 0.00 | 5.49 (226) 0.04 | 5.68 (218) 0.01 | 5.81 (213) 0.00 |
| AEtm8 | 4.27 (290) 0.18 | 4.75 (261) 0.00 | 5.48 (226) 0.04 | 5.68 (218) 0.01 | 5.83 (213) 0.00 |
| AIcm8 | 4.39 (282) 0.15 | 4.99 (248) 0.00 | 5.72 (217) 0.10 | 5.72 (217) 0.01 | 5.93 (209) 0.20 |
| AItm8 | 4.46 (278) 0.16 | 5.06 (245) 0.00 | 5.73 (217) 0.10 | 5.74 (216) 0.01 | 6.06 (204) 0.00 |
| 7m-8AG | | | | | |
| A1m7 | 4.40 (282) 0.12 | 5.06 (245) 0.00 | 5.33 (233) 0.00 | 5.53 (224) 0.18 | 5.66 (219) 0.01 |
| A3m7 | 4.65 (266) 0.00 | 4.95 (250) 0.19 | 5.37 (231) 0.16 | 5.56 (223) 0.00 | 5.90 (210) 0.00 |
| AEcm7 | 4.29 (289) 0.10 | 4.89 (254) 0.00 | 5.31 (233) 0.01 | 5.59 (222) 0.02 | 5.67 (219) 0.01 |
| AEtm7 | 4.26 (291) 0.10 | 4.82 (257) 0.00 | 5.24 (237) 0.01 | 5.57 (222) 0.03 | 5.67 (219) 0.01 |
| AIcm7 | 4.26 (291) 0.07 | 5.02 (247) 0.00 | 5.53 (224) 0.00 | 5.69 (218) 0.01 | 5.74 (216) 0.09 |
| AItm7 | 4.36 (285) 0.08 | 5.09 (244) 0.00 | 5.61 (221) 0.00 | 5.72 (217) 0.01 | 5.76 (215) 0.11 |

**Table S5**. The same as Table S2, but for ionic forms of natural and methylated 8AG.

| Form | S1 | S2 | S3 | S4 | S5 |
| --- | --- | --- | --- | --- | --- |
| B3LYP | | | | | |
| A1 (ma) | 4.58 (271) 0.12 | 4.84 (256) 0.00 | 5.02 (247) 0.00 | 5.15 (241) 0.09 | 5.20 (238) 0.00 |
| A (da) | 4.26 (291) 0.00 | 4.60 (270) 0.19 | 4.76 (260) 0.00 | 4.93 (252) 0.00 | 4.99 (249) 0.00 |
| Am9 (ma) | 4.47 (277) 0.25 | 4.66 (266) 0.00 | 4.87 (255) 0.00 | 5.05 (246) 0.03 | 5.10 (243) 0.00 |
| Am8 (ma) | 4.14 (300) 0.19 | 4.36 (284) 0.00 | 4.82 (257) 0.00 | 4.83 (257) 0.01 | 4.88 (254) 0.08 |
| Am7 (ma) | 4.18 (297) 0.12 | 4.51 (275) 0.00 | 4.79 (259) 0.00 | 4.97 (250) 0.01 | 5.00 (248) 0.03 |
| M06 | | | | | |
| A1 (ma) | 4.34 (286) 0.00 | 4.73 (262) 0.13 | 4.90 (253) 0.00 | 4.97 (250) 0.01 | 5.00 (248) 0.00 |
| A (da) | 3.79 (327) 0.00 | 4.30 (288) 0.00 | 4.38 (283) 0.00 | 4.57 (271) 0.01 | 4.74 (262) 0.18 |
| Am9 (ma) | 4.38 (283) 0.00 | 4.38 (270) 0.25 | 4.74 (261) 0.00 | 4.75 (261) 0.00 | 4.92 (252) 0.00 |
| Am8 (ma) | 4.26 (291) 0.21 | 4.35 (285) 0.00 | 4.43 (280) 0.00 | 4.60 (270) 0.00 | 4.93 (251) 0.00 |
| Am7 (ma) | 4.29 (289) 0.12 | 4.30 (289) 0.00 | 4.56 (272) 0.00 | 4.58 (271) 0.00 | 4.89 (254) 0.00 |
| PBE0 | | | | | |
| A1 (ma) | 4.76 (260) 0.14 | 5.08 (244) 0.00 | 5.17 (240) 0.00 | 5.32 (233) 0.10 | 5.37 (231) 0.00 |
| A (da) | 4.47 (278) 0.00 | 4.78 (259) 0.21 | 4.97 (249) 0.00 | 5.09 (243) 0.00 | 5.19 (239) 0.00 |
| Am9 (ma) | 4.69 (265) 0.27 | 4.86 (255) 0.00 | 5.10 (243) 0.00 | 5.27 (235) 0.01 | 5.34 (232) 0.00 |
| Am8 (ma) | 4.34 (286) 0.21 | 4.56 (272) 0.00 | 5.05 (245) 0.00 | 5.08 (244) 0.01 | 5.14 (241) 0.08 |
| Am7 (ma) | 4.38 (283) 0.13 | 4.71 (263) 0.00 | 5.01 (248) 0.00 | 5.24 (237) 0.02 | 5.26 (236) 0.02 |

**Table S6**. Vertical excitation and emission energies (eV) of selected low-energy tautomers and their methylated forms in water solution (raw, non-shifted data). All calculations were performed with the aug-cc-pvdz basis set. The oscillator strengths are provided in parentheses. The values best matching the experimental data are marked in the boldface type. The Mean Absolute Error (MAE) is reported in the last line. All absorption energies are provided in ESI. nc – not converged; nd – no data; ma – monoanion; da – dianion.

| Compound | | Absorption energy $\boldsymbol{E}^{\boldsymbol{vert-a}}$ (eV) | | | | | | Emission energy $\boldsymbol{E}^{\boldsymbol{vert-f}}$ (eV) | | | |
| --- | --- | --- | --- | --- | --- | --- | --- | --- | --- | --- | --- |
|  |  | B3LYP | | M06 | PBE0 | | Exp | B3LYP | M06 | PBE0 | Exp |
| NATURAL | A17 | 4.18 (0.11) | | 4.33 (0.12) | 4.38 (0.12) | | 4.98 | 2.90 | **3.15** | 3.17 | 3.14 |
|  | A18 | 4.24 (0.11) | | 4.54 (0.12) | 4.44 (0.12) | |  | 2.62 | 2.97 | 2.94 |  |
|  | A19 | 4.70 (0.16) | **5.02** | 4.89 (0.17) | 4.90 (0.16) | 5.17 |  | 3.35 | 3.64 | 3.61 |  |
|  |  | 5.14 (0.24) |  |  | 5.33 (0.27) |  |  |  |  |  |  |
| ANION | A1 | **4.58 (0.12)** | | 4.73 (0.13) | 4.76 (0.14) | | 4.46 | 3.36 | 2.73 | **3.55** | 3.49 |
|  | A3 | 4.87 (0.26) | | 5.01 (0.26) | 5.05 (0.28) | |  | 1.06 | 2.74 | 3.92 |  |
|  | A (da) | **4.59 (0.19)** | | 4.74 (0.18) | 4.78 (0.21) | | 4.43 | 2.22 | **3.22** | 2.70 | 3.44 |
| METHYL | A1M7 | **4.20 (0.11)** | | 4.34 (0.12) | 4.40 (0.12) | | 4.17 | 2.94 | 3.14 | **3.17** | 3.18 |
|  | A1M8 | **4.26 (0.15)** | | 4.42 (0.17) | 4.46 (0.17) | | 4.23 | 2.77 | **3.08** | 3.05 | 3.10 |
|  | A1M9 | 4.64 (0.17) | **4.96** | 4.84 (0.20) | 4.84 (0.18) | 5.17 | 4.94 | 3.35 | 3.61 | **3.59** | 3.54 |
|  |  | 5.12 (0.21) |  |  | 5.30 (0.23) |  |  |  |  |  |  |
| MET. ANIONS | AM9 (ma) | **4.47 (0.25)** | | 4.38 (0.25) | 4.69 (0.27) | | 4.48 | **3.48** | 2.72 | 3.70 | 3.43 |
|  | AM8 (ma) | **4.14 (0.19)** | | 4.26 (0.21) | 4.34 (0.21) | | 4.17 | 2.85 | 3.12 | **3.09** | 3.02 |
|  | AM7 (ma) | **4.18 (0.12)** | | 4.29 (0.12) | 4.38 (0.13) | | 4.13 | 2.76 | 3.02 | **3.04** | 3.06 |
| NUCLEOSIDE | | 4.88 (0.23) | 5.10 | nc | 4.88 (0.23) | 5.06 | nd | 3.59 | nc | 3.59 | nd |
|  |  | 5.31 (0.23) |  |  | 5.24 (0.23) |  |  |  |  |  |  |
| MAE (eV) | | 0.05 | | 0.17 | 0.24 | |  | 0.32 | 0.22 | 0.16 |  |

**Table S7.** The same as Table S6, but the original computed values are corrected by the optimal value of the shift determined for each method (Avila Ferrer et al. 2013; Bai et al. 2020).

| Compound | | Absorption energy $\boldsymbol{E}^{\boldsymbol{vert-a}}$ (eV) | | | | | | Emission energy $\boldsymbol{E}^{\boldsymbol{vert-f}}$ (eV) | | | |
| --- | --- | --- | --- | --- | --- | --- | --- | --- | --- | --- | --- |
|  |  | B3LYP | | M06 | PBE0 | | Exp | B3LYP | M06 | PBE0 | Exp |
| Shift (eV) | | -0.03 | | -0.18 | -0.23 | | - | 0.25 | 0.04 | 0.01 | - |
| NATURAL | A17 | 4.15 (0.11) | | 4.15 (0.12) | 4.15 (0.12) | | 4.98 | **3.15** | 3.19 | 3.18 | 3.14 |
|  | A18 | 4.21 (0.11) | | 4.36 (0.12) | 4.21 (0.12) | |  | 2.87 | 3.01 | 2.95 |  |
|  | A19 | 4.67 (0.16) | **4.99** | 4.71 (0.17) | 4.67 (0.16) | 4.94 |  | 3.50 | 3.68 | 3.62 |  |
|  |  | 5.11 (0.24) |  |  | 5.10 (0.27) |  |  |  |  |  |  |
| ANION | A1 | 4.55 (0.12) | | 4.55 (0.13) | **4.53 (0.14)** | | 4.46 | 3.61 | 2.77 | **3.56** | 3.49 |
|  | A3 | 4.84 (0.26) | | 4.83 (0.26) | 4.82 (0.28) | |  | 1.31 | 2.78 | 3.93 |  |
|  | A (da) | 4.56 (0.19) | | 4.56 (0.18) | **4.55 (0.21)** | | 4.43 | 2.47 | **3.26** | 2.71 | 3.44 |
| METHYL | A1M7 | **4.17 (0.11)** | | 4.16 (0.12) | **4.17 (0.12)** | | 4.17 | 3.19 | **3.18** | **3.18** | 3.18 |
|  | A1M8 | **4.23 (0.15)** | | 4.24 (0.17) | **4.23 (0.17)** | | 4.23 | 3.02 | **3.12** | 3.06 | 3.10 |
|  | A1M9 | 4.61 (0.17) | 4.93 | 4.66 (0.20) | 4.61 (0.18) | **4.94** | 4.94 | **3.60** | 3.65 | **3.60** | 3.54 |
|  |  | 5.09 (0.21) |  |  | 5.07 (0.23) |  |  |  |  |  |  |
| MET. ANIONS | AM9 (ma) | 4.44 (0.25) | | 4.20 (0.25) | **4.46 (0.27)** | | 4.48 | 3.73 | 2.76 | **3.71** | 3.43 |
|  | AM8 (ma) | **4.11 (0.19)** | | 4.08 (0.21) | **4.11 (0.21)** | | 4.17 | **3.10** | 3.16 | **3.10** | 3.02 |
|  | AM7 (ma) | **4.15 (0.12)** | | 4.11 (0.12) | **4.15 (0.13)** | | 4.13 | 3.01 | **3.06** | 3.05 | 3.06 |
| NUCLEOSIDE | | 4.85 (0.23) | 5.07 | nc | 4.60 (0.23) | 4.83 | nd | 3.84 | Nc | 3.60 | nd |
|  |  | 5.28 (0.23) |  |  | 5.01 (0.23) |  |  |  |  |  |  |
| MAE (eV) | | 0.04 | | 0.13 | 0.04 | |  | 0.19 | 0.22 | 0.16 |  |
